# “Signet Ring Sign” on Plain X-ray Indicates the Need for Surgical Intervention After Magnet Ingestion in Children

**DOI:** 10.7759/cureus.65943

**Published:** 2024-08-01

**Authors:** Lohit Sodagum, Paul Truche, Sathyaprasad Burjonrappa

**Affiliations:** 1 Pediatric Surgery, Rutgers Robert Wood Johnson Medical School, New Brunswick, USA

**Keywords:** enteric fistula, biliary-enteric fistula, surgery, abdominal foreign body, plain x-ray, multiple magnet ingestion

## Abstract

The ingestion of magnets used in toys and household products is a common problem in children and can have potentially devastating health consequences. The attraction between multiple magnets across intestinal walls can lead to bowel obstruction, fistula formation, necrosis, and perforation of the involved segments. Multiple magnets attached to each other within the intestinal lumen can also pass spontaneously. Clinical and radiological findings help guide the clinician in deciding whether to intervene surgically or follow an expectant management plan. We report the radiological finding of a "signet ring" on a plain abdominal X-ray that was associated with the surgical finding of fistula formation in two patients, who had clinically benign exams after magnet ingestion. This finding on plain abdominal X-rays should warrant operative exploration in children after magnet ingestion.

## Introduction

The ingestion of magnets, used in toys and modern household products, is a common occurrence in children and can have potentially devastating health consequences. Young children are at high risk of foreign body ingestion due to their tendency to put objects in their mouths and their less-developed chewing and swallowing ability [1]. In the 2022 Annual Report of the National Poison Data System from America’s Poison Centers, the ingestion of foreign bodies and toys was one of the top five most common exposures in children aged five years or less [2]. The National Electronic Injury Surveillance System estimated that there were 16,386 possible magnet ingestions among children aged <18 years from 2002 to 2011 in the United States [3]. The majority of children who ingest magnets are less than five years of age and are male [1]. Children who present with single-magnet ingestions are often asymptomatic and have a faster passage of the magnets than those who ingest multiple magnets [4]. The ingestion of multiple magnets is worrisome as the attraction between magnets in distinct segments of the bowel can lead to fistula formation, bowel obstruction, volvulus, necrosis, and perforation [5]. However, due to the possibility of spontaneous passage of multiple magnets that remain connected, these patients are often observed with serial radiographic imaging rather than treated with surgical intervention. The aim of this case series is to describe two patients who presented with distinct radiological findings of a ringlike structure after the ingestion of multiple magnets and to increase awareness of the need for high clinical suspicion for a fistulous process when presented with these findings.

This manuscript was prepared following the CARE guidelines (https://www.care-statement.org).

## Case presentation

Case 1

Case 1 was a 17-month-old male toddler transferred to our institution from an outside facility with foreign body ingestion reported to have occurred approximately five hours earlier. The object was believed to be a small toy. The patient had been unwilling to eat or drink and had discomfort with attempts to do so, although he had no cough or respiratory difficulties. He was noted to be actively drooling upon exam in the emergency department.

**Figure 1:**
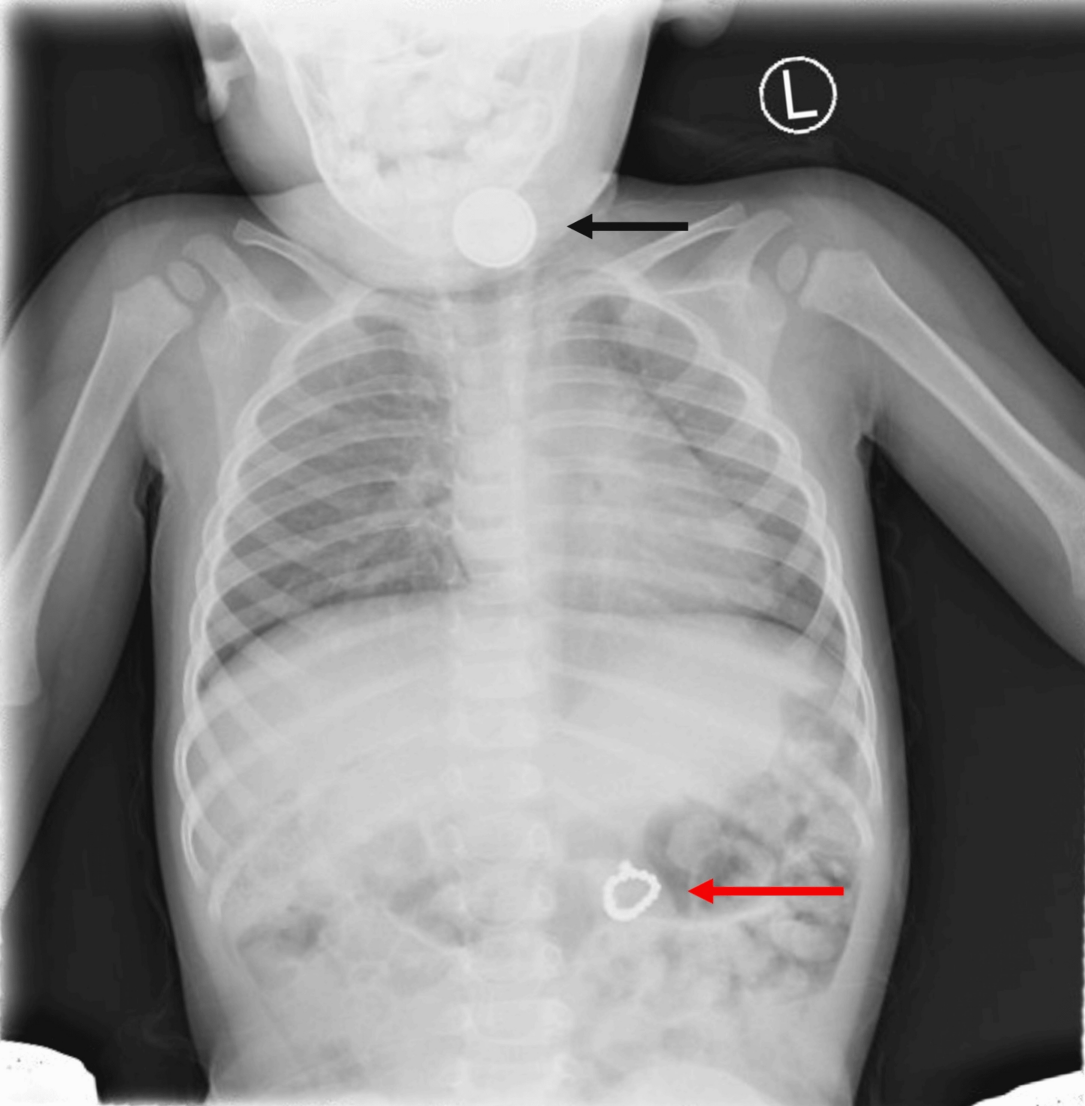
X-ray demonstrating a 'halo sign' (black marker) from a button battery impacted in the cervical esophagus and a "signet ring" sign (red marker) from multiple magnets causing a gastrojejunal fistula in the stomach

Chest X-ray performed at the outside facility demonstrated two visible foreign bodies: a 20 mm dense disc-like foreign body, coronally oriented at the level of the lower cervical esophagus, and in the left upper quadrant, likely the left side of the transverse colon, a second ringlike foreign body approximately 15 mm in diameter, composed of multiple 2.5 mm beads, with an additional slightly greater than 2.5 mm irregular density attached at its upper margin. There was no obstruction associated with either of the foreign bodies noted on imaging (Figure 1). 

Chest and abdominal X-rays performed at our institution confirmed the findings of the previous imaging. The appearance of the foreign body in the esophagus was concerning for a button battery. The patient was taken for an emergency rigid esophagoscopy and a lithium button battery was removed, with inflammation of the superficial mucosa of the proximal esophagus noted. The object was ultimately identified as a Maxell CR2025 3V lithium battery. The patient was admitted to the surgical service with plans to monitor the progression of the magnets.

**Figure 2:**
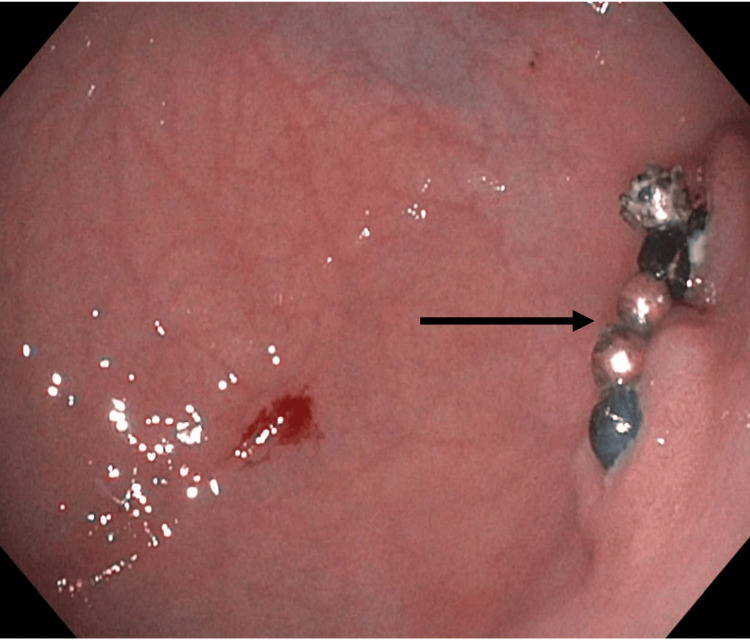
Endoscopic image of half of the ring of magnets (black marker) partially embedded in the mucosa of the gastric antrum

On the following day, the patient had a repeat abdominal X-ray, which demonstrated no significant interval change in position. Given the lack of progression, the patient was taken back to the OR, where an attempt at endoscopic removal was made. On endoscopy, multiple magnets had formed a fistula between the stomach and intestine. Half of the ring of magnets was identified in the antrum, partially embedded in the gastric mucosa (Figure 2). The visible magnetic beads were mobilized by water flush and removed with a Roth Net retriever.

**Figure 3:**
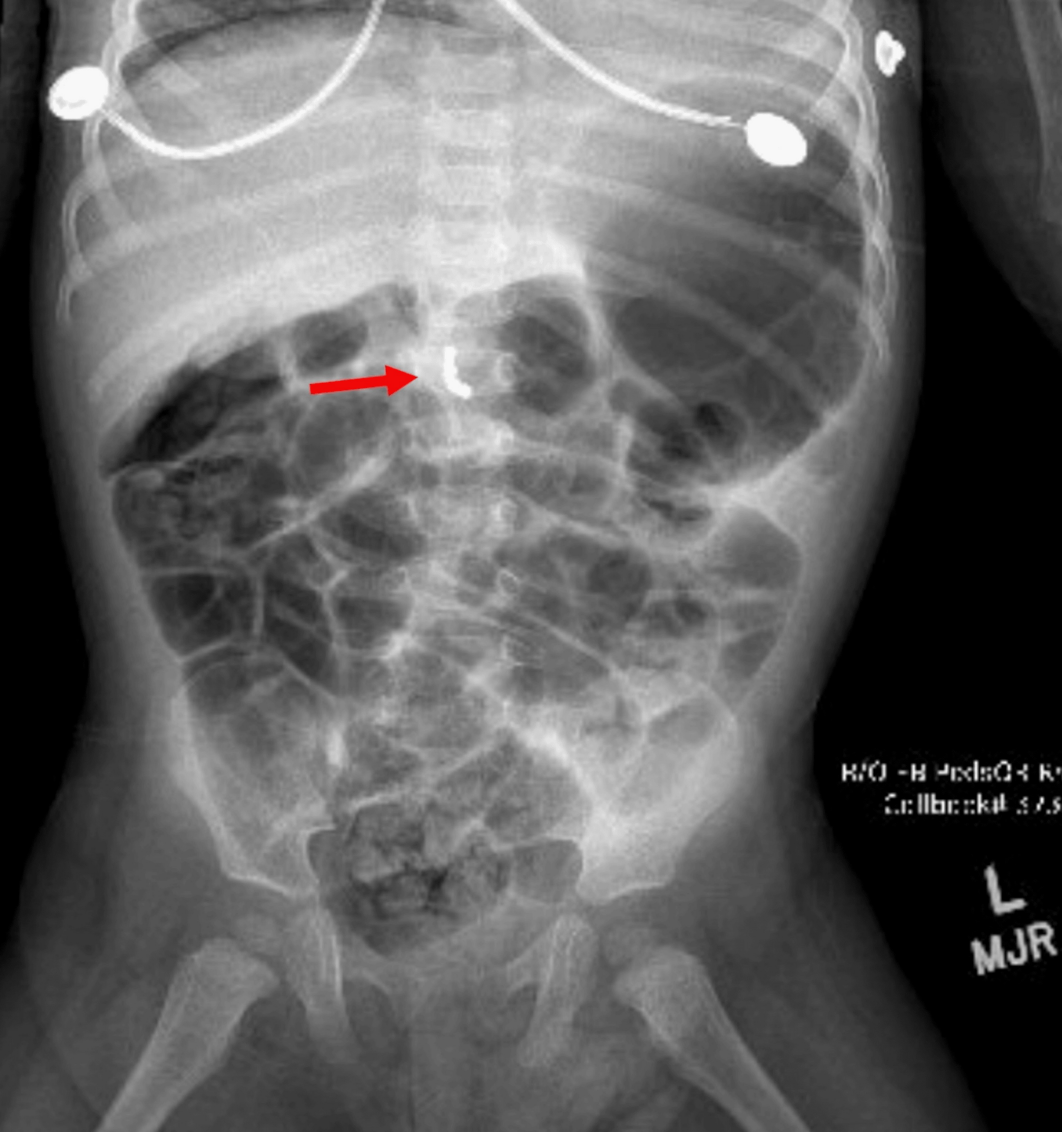
Intraoperative X-ray demonstrating magnetic beads (red marker) in the duodenum

**Figure 4:**
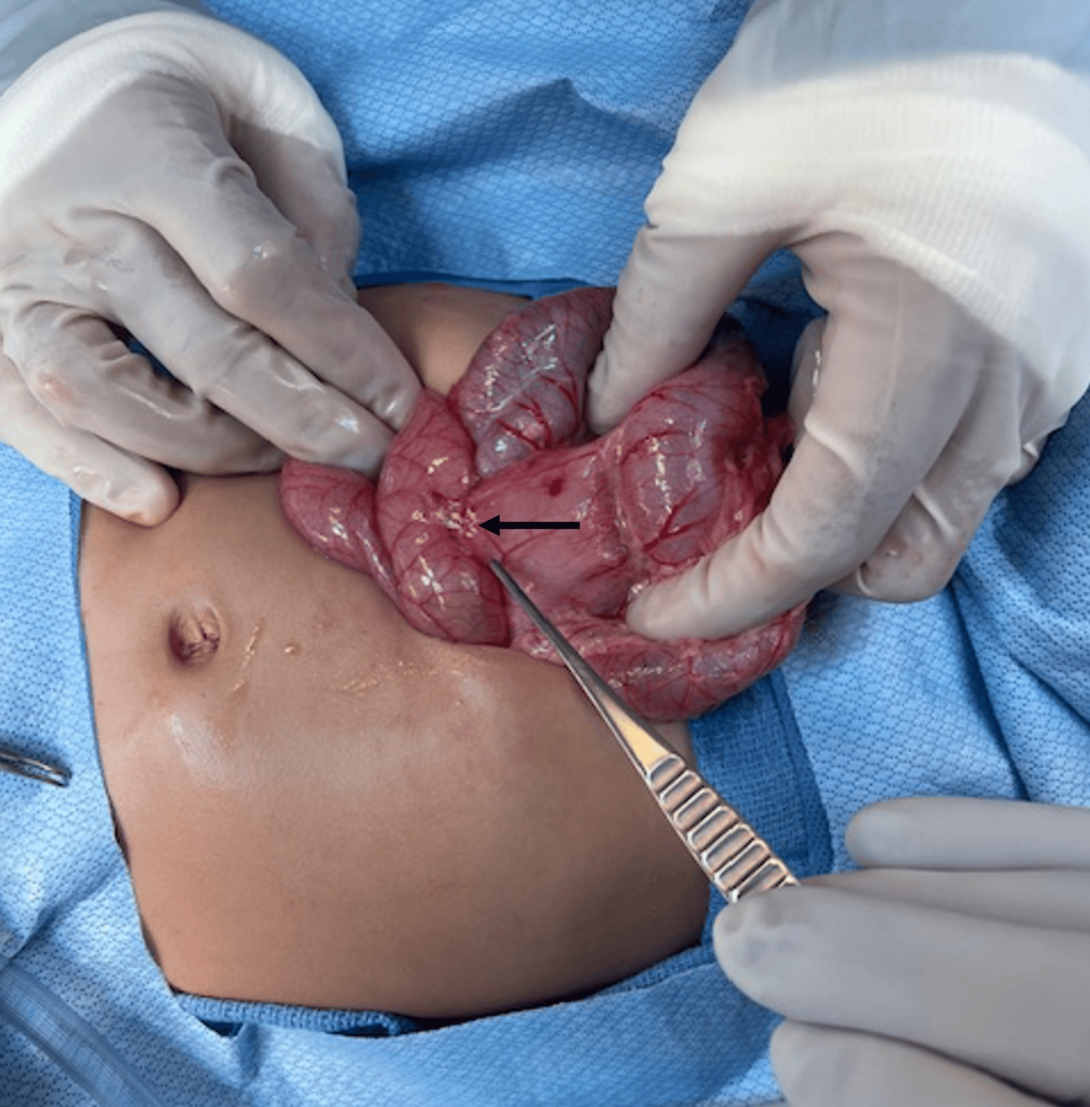
Intraoperative finding of the gastrojejunal fistula (black marker)

Intraoperative X-ray identified additional magnetic beads in the duodenum, leading to an additional attempt at endoscopic removal (Figure 3). Not all the magnets could be visually identified on endoscopy. Given these findings, the patient underwent an exploratory laparotomy. The lesser sac was exposed by taking down the gastrocolic and gastrosplenic ligaments. A fistulous communication was identified between the stomach and the proximal jejunum across the transverse mesocolon (Figure 4). The enterocutaneous fistula was taken down, and the stomach was closed primarily. The jejunum had two additional fistulous sites adjacent to each other. They were connected by opening the bridge of the intact small bowel and subsequent primary repair.

**Figure 5:**
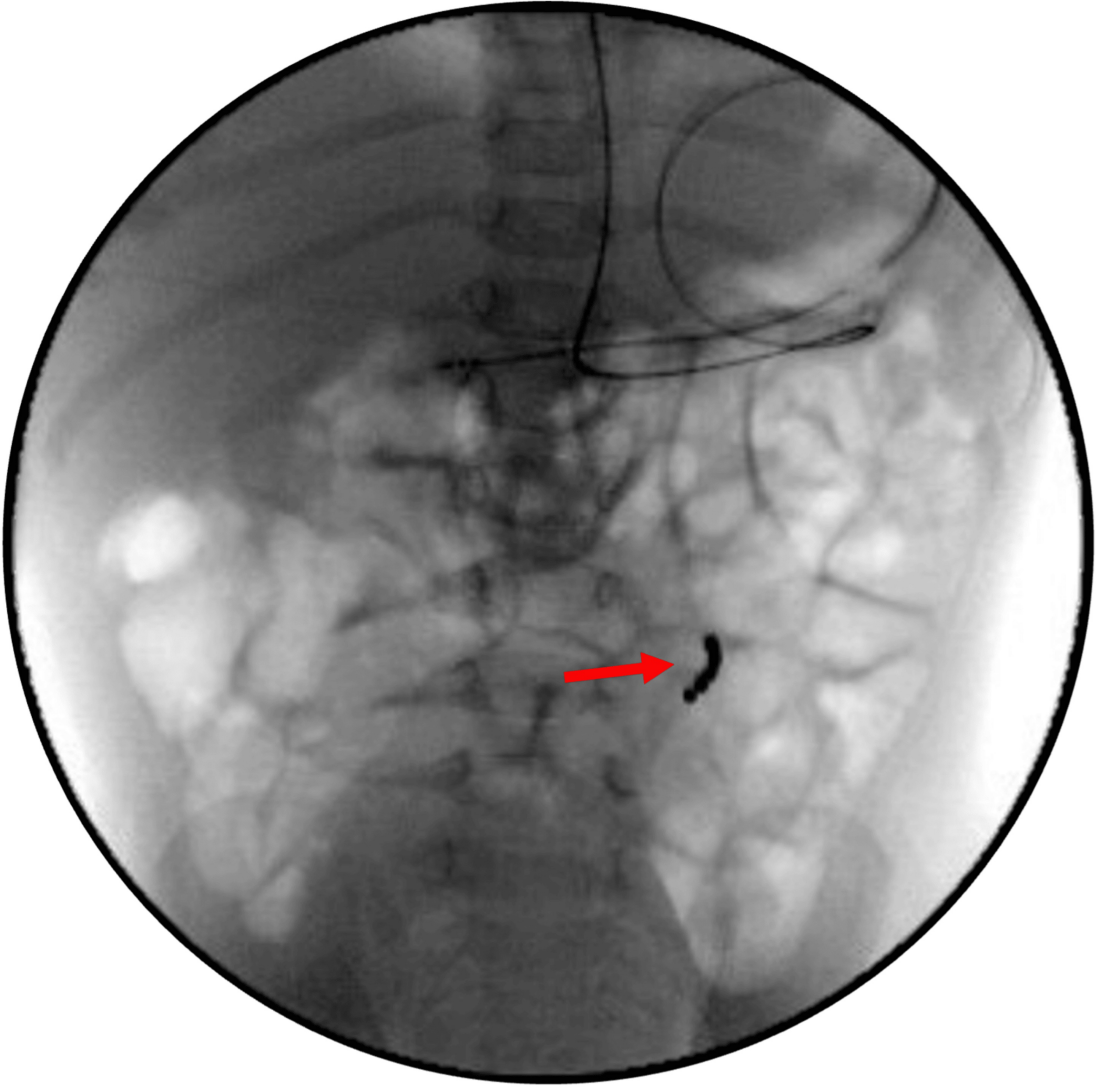
Intraoperative fluoroscopy demonstrating magnets in the small bowel (red marker)

**Figure 6:**
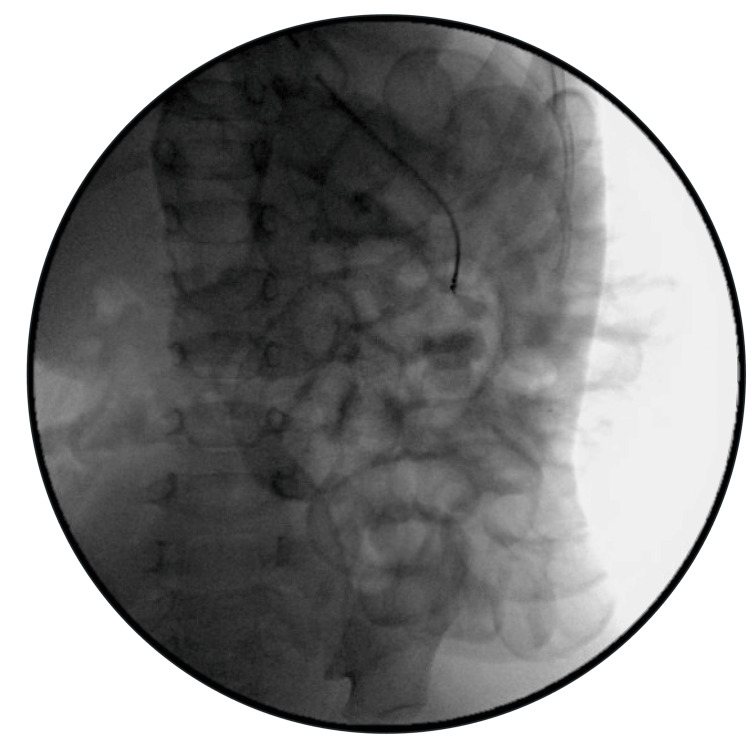
Intraoperative fluoroscopy demonstrating no further magnets remaining in the abdomen after surgical removal

After repair, the bowel was examined from proximal to distal, and no foreign body was initially identified. Intraoperative fluoroscopy was obtained, which identified the foreign body in a segment of the small bowel (Figure 5). A sterile pacemaker magnet was used to scan the bowel, and the metallic foreign bodies were identified. Six magnets were stuck to each other and a small enterotomy was made at the mid-small bowel to express these magnets. Fluoroscopy was performed again and confirmed that there were no further magnets left in the abdomen (Figure 6). The patient was discharged on postoperative day (POD) four and seen 10 days later for a post-operative follow-up, at which time he was recovering without complications.

Case 2

Case 2 was a five-year-old male child who presented to the emergency department with three to four episodes of non-bloody, non-bilious vomiting throughout the day, as well as right-sided abdominal pain. Prior to these episodes, the patient had one week of cyclical abdominal pain associated with nausea and vomiting. His parents also described a low-grade fever. He had otherwise been tolerating a regular diet and having regular bowel function. He had recently been treated for recurrent strep throat and had just finished his course of oral antibiotics the day prior to the presentation. The patient’s past medical history was significant for a bicuspid aortic valve that had not required surgical intervention. He had no previous intra-abdominal surgeries.

**Figure 7:**
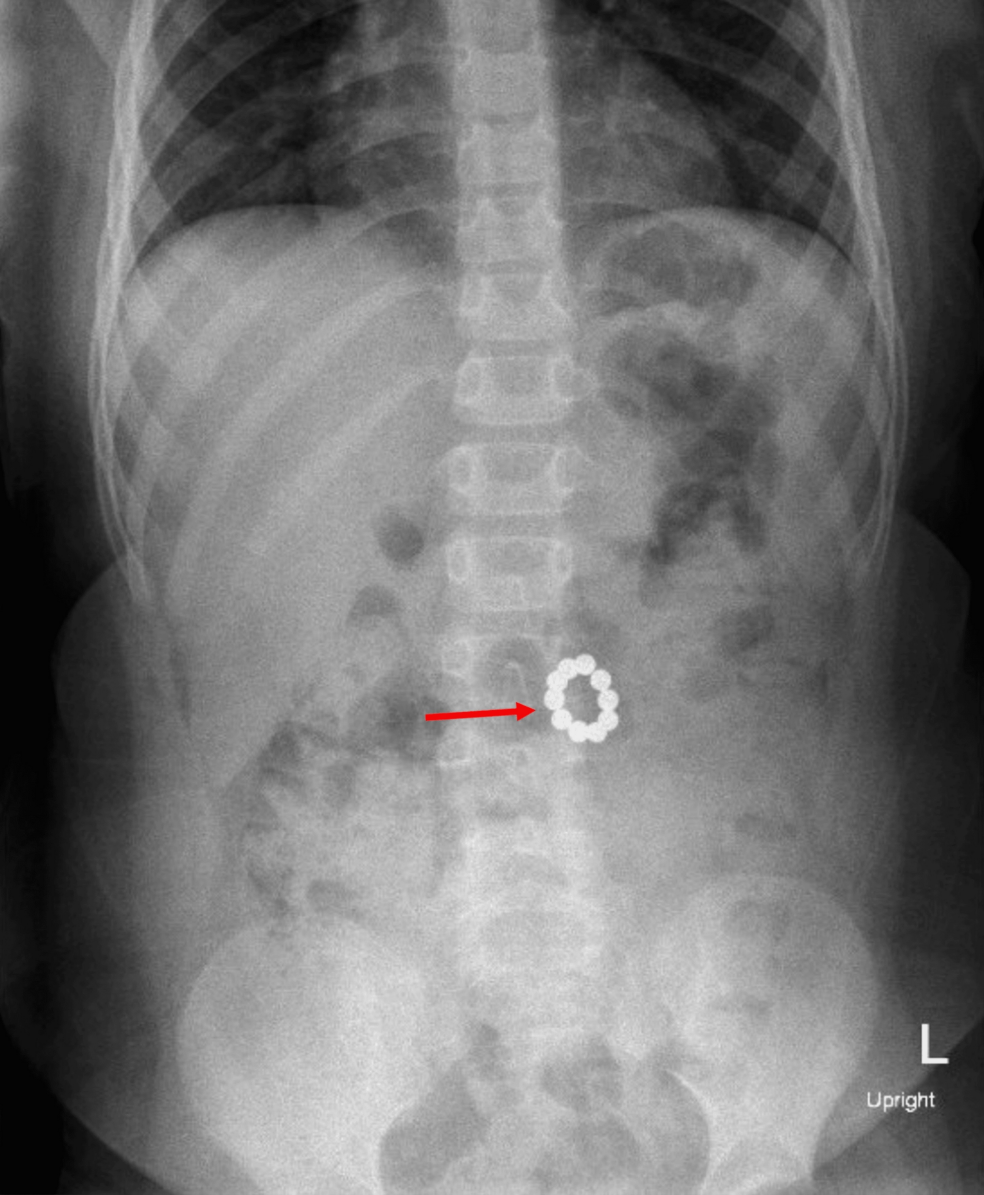
X-ray demonstrating the formation of a "signet ring" (red marker) in the bowel after ingestion of multiple magnets

An abdominal X-ray revealed a ring made up of 10 small magnets that appeared to be in the transverse colon (Figure 7). The patient was admitted for serial X-rays to assess the progression of the foreign body. On repeat abdominal X-ray the following morning, the magnets showed no progression or change in position. Given the ring-like appearance of the magnets, there was high clinical suspicion of a fistulous process, and the patient underwent an exploratory laparotomy through a transverse right upper quadrant incision. The small bowel was evaluated from the ligament of Treitz. Magnets were immediately visualized partially protruding through the bowel wall with three perforations just distal to the ligament of Treitz. The magnets were delivered through the site of perforation using mechanical manipulation and a pacemaker magnet.

**Figure 8:**
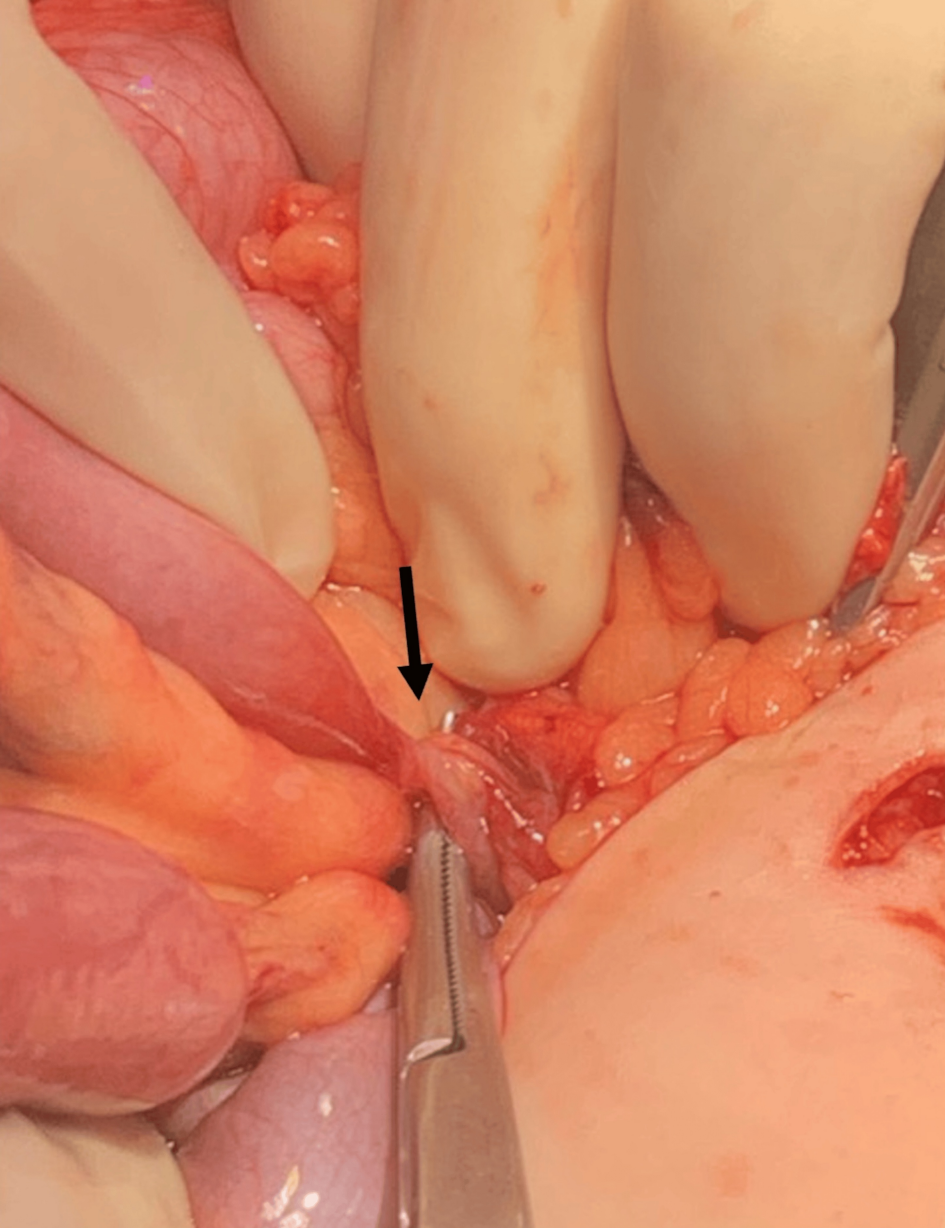
Intraoperative finding of a jejunocolic fistula (black marker), approximately 30 cm distal to the ligament of Treitz

A second perforation was noted close to the mesenteric border of the jejunum, approximately 30 cm distal to the ligament of Treitz, but without any magnets in proximity, suggesting a fistulated tract. At this location, the bowel was also tethered to the sigmoid colon (Figure 8). The area of the small bowel was resected, and the colotomy was repaired primarily. The resected bowel specimen was opened, and no magnets were identified. 

On POD six, the patient developed a superficial infection of the surgical site and was treated with bedside incision and drainage and five days of intravenous piperacillin-tazobactam. The patient was discharged on POD 12 and seen in the outpatient office six days later with persistent serous drainage from the incision. The patient was prescribed an extended antibiotic course of ciprofloxacin and metronidazole for five days to treat the abscess. He returned to the office one week later, at which time the incision was healing without drainage or erythema.

## Discussion

Multiple-magnet ingestion in pediatrics is a serious issue that can lead to severe complications. Magnets are attractive to children due to their small size and shiny appearance, making them easy to swallow accidentally. When multiple magnets are ingested, they can attract to each other through different loops of the gastrointestinal (GI) tract, leading to fistula formation and perforation.

In recent years, there has been an increase in reported cases of multiple-magnet ingestion, often involving high-powered neodymium magnets. Neodymium magnets are five to 30 times stronger than conventional magnets and are sold as popular toys and household objects, including Buckyballs and the NeoCube [6,7]. These magnets are particularly concerning because of their strong attractive force and their small size, which facilitates passage through the GI tract.

Flaherty et al. described trends in emergency department visits for ingestions of small neodymium magnets between 2009 and 2019, before, during, and after a 2014 Consumer Product Safety Commission (CPSC) rule restricting their sales. Between 2012 and 2016, there was a decline in visits for magnet ingestions, suggesting that this legislature did reduce magnet ingestion. Following the removal of the rule in 2016, there was a resultant increase in emergency department visit rates from 2017 to 2019 [8]. 

The clinical presentation of multiple-magnet ingestion can vary, ranging from asymptomatic to life-threatening. Symptoms may include abdominal pain, nausea, vomiting, and signs of GI obstruction [9]. In Case 1, the patient had ingested a button battery and multiple magnets and presented with reduced oral intake, increased drooling, and a benign abdominal exam. In Case 2, the patient had one week of cyclical abdominal pain and vomiting but was not in acute distress on hospital admission. Diagnosis becomes challenging in cases where the patients are asymptomatic. Case 1 illustrates the importance of thoroughly evaluating imaging findings as the magnets were incidentally detected in the bowel on a chest X-ray that was performed to evaluate drooling and dysphagia that identified the button battery. Multiple foreign body ingestion must be considered in children, and these may not be in the same location in the gastrointestinal tract.

Management of multiple-magnet ingestion involves prompt identification and removal of the magnets. The 2015 North American Society for Pediatric Gastroenterology, Hepatology, and Nutrition (NASPGHAN) guidelines for the management of magnet ingestions state that multiple magnets still in the stomach can be removed endoscopically, and if beyond the stomach, it should be managed according to symptoms and progression. In asymptomatic patients with no obstruction or perforation on X-ray, the progression of the magnets may be followed with serial X-rays. Symptomatic patients should be referred for surgical intervention [7]. While non-progression of magnets is an absolute indication for surgical presentation, we propose that the presence of a "signet ring" on abdominal X-ray, in the setting of magnet ingestion, is in itself an indication for surgical intervention. This happens only in the instance of fistulation across different segments of the gastrointestinal tract and will not happen if all the magnets are within the same luminal location.

We propose that patients presenting with the formation of a "signet ring" by multiple magnets be referred for prompt surgical intervention. In the cases described, the ring of magnets appeared to be intact and within a single lumen of the bowel on X-ray, but direct visualization during surgery revealed bowel perforation and fistulous connections.

There are additional case reports that describe patients with minimal symptoms presenting with the formation of a ring of magnets and were discovered during surgery to have developed intestinal perforations and fistulas [10,11]. Our case series adds to the growing body of literature on pediatric foreign body ingestion by detailing a unique radiographic finding that should increase suspicion of complications regardless of presenting symptoms. Ultimately, children with a ring of magnets on imaging warrant high suspicion of fistula and perforation and should be followed closely with a low threshold to operate.

## Conclusions

Multiple-magnet ingestion among children is a serious issue that requires prompt recognition and management. The presented cases highlight the need for a high clinical suspicion for a fistulous process when a "signet ring" is encountered on a plain abdominal X-ray. This radiological finding in itself warrants surgical intervention precluding the need for repeat X-rays to document failure to progress. These patients require early surgical intervention, regardless of symptomatology. Furthermore, it is important to thoroughly evaluate radiological findings so as to not miss multiple foreign bodies. The prevention of magnet ingestion is crucial in reducing the incidence of this potentially fatal problem. Caregivers should be educated about the dangers of magnets and the importance of keeping them out of reach of children.

## References

[1] Abbas MI, Oliva-Hemker M, Choi J (2013). Magnet ingestions in children presenting to US emergency departments, 2002-2011. J Pediatr Gastroenterol Nutr.

[2] Gummin DD, Mowry JB, Beuhler MC (2023). 2022 Annual Report of the National Poison Data System(®) (NPDS) from America's Poison Centers(®): 40th Annual Report. Clin Toxicol (Phila).

[3] Alfonzo MJ, Baum CR (2016). Magnetic foreign body ingestions. Pediatr Emerg Care.

[4] Alansari AN, Baykuziyev T, Soyer T (2024). Magnet ingestion in growing children: a multi-center observational study on single and multiple magnet incidents. Sci Rep.

[5] Han Y, Youn JK, Oh C, Lee S, Seo JM, Kim HY (2020). Ingestion of multiple magnets in children. J Pediatr Surg.

[6] Altokhais T (2021). Magnet ingestion in children management guidelines and prevention. Front Pediatr.

[7] Kramer RE, Lerner DG, Lin T (2015). Management of ingested foreign bodies in children: a clinical report of the NASPGHAN Endoscopy Committee. J Pediatr Gastroenterol Nutr.

[8] Flaherty MR, Buchmiller T, Vangel M, Lee LK (2020). Pediatric magnet ingestions after federal rule changes, 2009-2019. JAMA.

[9] Hussain SZ, Bousvaros A, Gilger M, Mamula P, Gupta S, Kramer R, Noel RA (2012). Management of ingested magnets in children. J Pediatr Gastroenterol Nutr.

[10] Feng Ji Mervin G, Ali AF, Kheng Lincoln Dale LS, Vidyadhar M (2020). Multiple magnet ingestion: ring-like configuration with multiple intestinal fistulae. J Pediatr Surg Case Rep.

[11] Mili T, Charieg A, Ahmed YB, Marzouki M, Nouira F, Jlidi S (2023). A case report of multiple foreign body ingestion with a ring-like configuration: The magnetic effect. Int J Surg Case Rep.

